# Prevalence of Episiotomy Practice and Associated Factors in Brazil: A Systematic Review

**DOI:** 10.1111/birt.70073

**Published:** 2026-05-04

**Authors:** Ana Carolina dos Santos Rodrigues, Maria Luziene de Sousa Gomes, Roger Rodrigues da Silva, Eremita Val Rafael, Patrícia Ribeiro Azevedo, Claudia Teresa Frias Rios, Janielle Ferreira de Brito Lima, Mônica Oliveira Batista Oriá

**Affiliations:** ^1^ Department of Nursing Federal University of Maranhão São Luís Maranhão Brazil; ^2^ Department of Nursing Federal University of Ceará Fortaleza Ceará Brazil

**Keywords:** episiotomy, humanizing delivery, natural childbirth, obstetric practice, women's health

## Abstract

**Introduction:**

Although routine episiotomy is not recommended, it remains widely performed during childbirth, with rates varying substantially across countries. This systematic review synthesizes evidence on the prevalence and associated factors of episiotomy in Brazil.

**Methods:**

This is a systematic review of prevalence, conducted in accordance with the recommendations of the Joanna Briggs Institute (JBI). The protocol was registered in the International Prospective Register of Systematic Reviews (PROSPERO) under the identification number CRD42024621526. Seven electronic databases were searched with no restrictions regarding publication period or language: MEDLINE/PubMed (via the National Library of Medicine), Web of Science, SCOPUS, CINAHL (EBSCO), Cochrane, EMBASE (Elsevier), and the Latin American and Caribbean Health Sciences Literature (LILACS). The JBI Critical Appraisal Checklist for Studies Reporting Prevalence Data was used by two authors to assess the quality of the selected studies.

**Results:**

A total of 65 studies were included. The prevalence of episiotomy practice among women varied from 0.5% to 94%. Only 16 studies (24.6%) reported episiotomy rates close to the recommended benchmark of 10%. Thirty‐three studies (50.8%) analyzed factors associated with the practice, with the most frequently reported factors being: primiparity, primigravidity, nulliparity, adolescence, youth, higher education, higher income, delivery in private healthcare settings, physician‐assisted births, use of oxytocin, instrumental delivery, absence of previous vaginal birth, prematurity, newborn weight over 4000 g, and horizontal delivery position.

**Conclusions:**

A limited number of studies have met recommended episiotomy rates, underscoring the challenge of translating evidence into practice. Lower rates were seen in births attended by nurse midwives. Continuous professional training focused on evidence‐based care is essential to reduce unnecessary interventions and align rates with recommended targets.

## 1 Introduction

Episiotomy is defined as the surgical enlargement of the vaginal opening through an incision in the perineum during the final part of the second stage of labor or delivery, aimed at increasing the diameter of the vaginal outlet to facilitate the birth of the baby [1, 2]. The literature describes seven types of episiotomy (median, modified median, J‐shaped, mediolateral, lateral, radical lateral or Schuchardt incision, and anterior) [1, 2].

Episiotomy rates vary considerably across countries on a global scale. Studies report that the prevalence of the procedure was 41.7% in Africa [3], 45.1% in Ethiopia [4], 20% in Indonesia [5], and 41.7% in China [6]. In low‐ and middle‐income countries, a systematic review involving 41 countries estimated a pooled episiotomy prevalence of 46%. However, this rate varies substantially across different regions and obstetric populations, often exceeding this average in primiparous women [7].

In Brazil, the scenario is characterized by significant heterogeneity depending on the region and the period analyzed. While recent cross‐sectional studies in specific hospital settings report prevalence rates ranging from 18.7% [8] to 26.3% [9], a major national survey identified a prevalence of 53.5% [10]. A study conducted in the Southern region demonstrated a significant reduction in the practice over the last decade, with rates dropping from 70.9% in 2007 to 19.4% in 2019 [11]. These findings highlight that, although there is a downward trend in some areas, the episiotomy rate in Brazil still remains above the World Health Organization (WHO) recommendation, which sets a reference rate of around 10% for vaginal deliveries [12].

In Brazil, the high prevalence of episiotomy stands in direct contrast to the movement for Humanized Childbirth, which advocates for respectful, evidence‐based maternity care. The procedure has become a central topic in the debate regarding reproductive rights and health policies. Although Brazilian National Guidelines explicitly advise against routine use, the practice persists, often reflecting a technocratic model of care [13]. According to the Brazilian National Guidelines for Normal Childbirth Care, episiotomy should not be routinely performed during spontaneous vaginal delivery; when indicated, effective analgesia must be provided prior to the procedure, and its execution must be properly justified and communicated to the woman in labor. The recommended technique, according to the guidelines, is the mediolateral incision originating at the vaginal fourchette and directed to the right side, with an angle of 45 to 60 degrees from the vertical axis [13].

In this context, the adoption of best practices for childbirth and delivery care, based on respectful and woman‐centered care, becomes essential [12]. The use of a specific informed consent form for episiotomy is considered essential [14], as performing the procedure without consent or in a forced manner constitutes a form of obstetric violence [15]. Crucially, performing episiotomy without the woman's explicit informed consent is increasingly recognized as a form of obstetric violence and a violation of bodily autonomy. Therefore, aligning practice with recommended benchmarks is not only a clinical necessity but an imperative for the protection of women's fundamental rights [15].

Although historically justified to prevent severe trauma, current evidence indicates that episiotomy is associated with significant maternal morbidity. Physical consequences include an increased risk of severe perineal lacerations (third‐ and fourth‐degree), infection, urinary incontinence, wound dehiscence, and chronic pain. Furthermore, the procedure can compromise sexual health, leading to long‐term dyspareunia and sexual dysfunction. Beyond physical sequelae, recent studies highlight the psychological toll, linking episiotomy to negative childbirth experiences, impaired body image, and adverse mental health outcomes, including post‐traumatic stress symptoms and postpartum depression [10, 11, 13, 14, 15].

While studies have addressed episiotomy in Brazil, the existing literature consists predominantly of regional investigations restricted to specific local contexts. This results in a fragmented body of evidence, characterized by significant heterogeneity and lacking consolidation. Consequently, a unified national panorama remains absent, hindering the assessment of the true magnitude of the problem and the formulation of effective public policies. To bridge this gap, this study aimed to synthesize the evidence on the prevalence and associated factors of episiotomy in Brazil.

## 2 Methods

This is a systematic review of prevalence, conducted in accordance with the recommendations of the Joanna Briggs Institute (JBI) [16]. The protocol was registered in the International Prospective Register of Systematic Reviews (PROSPERO) under the identification number CRD42024621526. The final report was prepared following the PRISMA guidelines [17] (File SS1).

This study was based on data that were previously published and are publicly available; therefore, it did not require submission to or approval by a Research Ethics Committee. It is important to highlight that the fundamental ethical principles of scientific research were followed, including methodological transparency, academic integrity, and respect for copyright.

### 2.1 Development of the Research Question

The research question was guided by the PICO strategy, considering: “P” (Population or Problem): Women who had vaginal deliveries in Brazil; “I” (Intervention): performance of episiotomy; “C” (Comparison): not applicable; and “O” (Outcome): prevalence of episiotomy and factors associated with its performance. Thus, the guiding question was: What is the prevalence of episiotomy in vaginal deliveries in Brazil, and what factors are associated with the performance of this intervention?

### 2.2 Eligibility Criteria

Studies were included in this review if they met all of the following criteria: (a) quantitative and observational studies (descriptive, cross‐sectional, cohort, case–control, ecological, and longitudinal); (b) studies reporting the prevalence of episiotomy, either as a primary or secondary outcome; (c) studies conducted with women in labor of all age groups and ethnicities; and (d) data on the Brazilian population, with no restrictions regarding the period of publication or language.

It is important to note that the reporting of associated factors was not a mandatory inclusion criterion. Studies were included if they provided prevalence data; information regarding associated factors was extracted and synthesized only when available in primary studies. Qualitative research, systematic reviews, opinion articles, experience reports, ongoing trials, incomplete articles, book chapters, editorials, and procedural reports were excluded.

### 2.3 Information Sources and Search Strategies

A systematic literature search was conducted on December 2, 2024, using the following data sources: MEDLINE/PubMed (via the National Library of Medicine), Web of Science, SCOPUS, CINAHL (EBSCO), Cochrane, EMBASE (Elsevier), and the Latin American and Caribbean Health Sciences Literature (LILACS) via the Virtual Health Library (BVS). The databases were accessed free of charge through the Portal de Periódicos of the Brazilian Coordination for the Improvement of Higher Education Personnel (CAPES).

Search strategies for electronic databases were developed using controlled descriptors indexed in Medical Subject Headings (MeSH) and Emtree. After defining the search terms, they were combined using the Boolean operators AND and OR. The search strategy used for each database is detailed in Table 1.

**TABLE 1:** Search strategies in electronic databases, 2024.

Databases	Search strategy
PubMed	(“Pregnant women” OR Pregnancy OR Women) AND (Episiotomy OR Episiotomies) AND (Brazil OR Brazilian OR Brasil)
Web of Science	
SCOPUS	
CINAHL	
Cochrane	
EMBASE	
LILACS	

*Note:* Table prepared by the authors.

### 2.4 Study Selection

The search results from each database were imported into the reference management software Rayyan for study organization, duplicate removal, selection, and screening [18]. Two reviewers independently screened the titles and abstracts of all references. Subsequently, the full texts of potentially eligible studies were independently assessed by both reviewers to determine whether all inclusion criteria were met. In case of disagreements, a third author resolved the selection discrepancies.

### 2.5 Data Extraction

Data from the included studies were extracted using a customized form, developed and tested by the authors. Data extraction was also conducted independently by two reviewers, considering the following variables: author, year of publication, and study region; study objective(s); method (study design, location, and period of execution); population/sample; prevalence of episiotomy; factors associated with episiotomy; and main outcomes.

### 2.6 Quality Assessment

The JBI Critical Appraisal Checklist for Studies Reporting Prevalence Data [16] was used by two authors to assess the quality of the selected studies.

### 2.7 Outcome Measurement

This review analyzed two primary outcomes. The first was the prevalence of episiotomy, defined as the surgical incision of the perineum and vaginal opening. The second outcome comprised the factors associated with the procedure. These encompassed maternal, sociodemographic, obstetric, clinical, and institutional characteristics that were statistically associated with or frequently reported in relation to episiotomy.

### 2.8 Data Synthesis and Analysis

The results were analyzed and synthesized both descriptively and quantitatively. The studies were grouped to provide an overview of the scope, nature, and distribution of the included literature. It is important to specify that the number of studies contributed differently to each outcome: the synthesis of prevalence included all eligible studies, whereas the synthesis of associated factors was restricted to the subset of studies that reported statistical associations for the procedure or frequently reported in relation to episiotomy. Tables and figures were used to present the extracted data, considering these distinct groups.

## 3 Results

**FIGURE 1:**
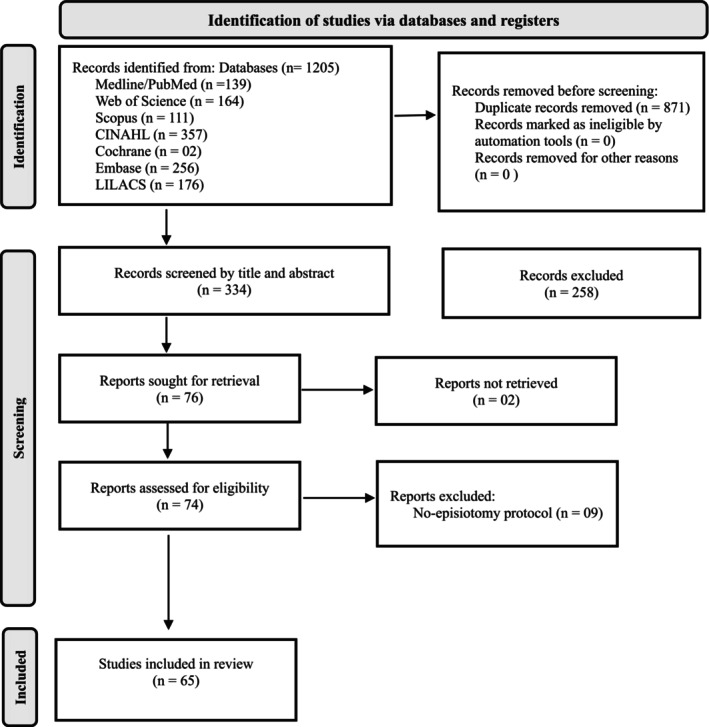
PRISMA flow diagram summary of article search, identification, and selection process.

A total of 1205 records were identified. After duplicate removal, 334 records remained for title and abstract screening. Of these, 258 records were excluded for not meeting the inclusion criteria, resulting in 76 studies selected for full‐text review. Two studies could not be retrieved. Additionally, nine articles were excluded for not meeting the inclusion criteria, and 65 articles were included in the review. The search and study selection process is detailed in Figure 1.

### 3.1 Methodological Quality

The included articles were assessed for methodological quality using the JBI Critical Appraisal Checklist for Studies Reporting Prevalence Data [16]. Table 2 provides a summary of the methodological quality of the included studies.

**TABLE 2:** Summary of Joanna Briggs Institute (JBI) critical appraisal checklist.

Author/Year	Q1	Q2	Q3	Q4	Q5	Q6	Q7	Q8	Q9	Total	%
Leal et al. 2014 [10]	Y	Y	Y	U	Y	Y	Y	Y	Y	8/9	88.8
César et al. 2022 [11]	Y	Y	Y	Y	Y	Y	Y	Y	Y	9/9	100
Medina et al. 2023 [19]	Y	Y	Y	Y	Y	Y	Y	Y	Y	9/9	100
Ferreira et al. 2023 [20]	Y	Y	Y	Y	Y	Y	Y	Y	Y	9/9	100
Rodrigues et al. 2022 [21]	Y	Y	Y	Y	Y	Y	Y	Y	Y	9/9	100
Albuquerque et al. 2022 [8]	Y	Y	Y	Y	Y	Y	Y	Y	Y	9/9	100
Nakata et al. 2022 [22]	Y	Y	Y	Y	Y	Y	Y	Y	Y	9/9	100
Pelissari et al. 2022 [23]	Y	Y	Y	Y	Y	Y	Y	Y	Y	9/9	100
Marmitt et al. 2022 [24]	Y	Y	Y	U	Y	Y	Y	Y	Y	8/9	88.8
Monteiro et al. 2021 [25]	Y	Y	Y	Y	Y	Y	Y	Y	Y	9/9	100
Domenighi et al. 2021 [26]	Y	Y	Y	Y	Y	Y	Y	Y	Y	9/9	100
Alcântara et al. 2021 [27]	Y	Y	Y	Y	Y	Y	Y	Y	Y	9/9	100
Angelim et al. 2021 [28]	Y	Y	Y	Y	Y	Y	Y	Y	Y	9/9	100
Alves et al. 2021 [29]	Y	Y	Y	U	Y	Y	Y	Y	Y	8/9	88.8
Gama et al. 2021 [30]	Y	Y	Y	U	Y	Y	Y	Y	Y	8/9	88.8
Souza et al. 2020 [31]	Y	Y	Y	Y	Y	Y	Y	Y	Y	9/9	100
Aguiar et al. 2020 [9]	Y	Y	Y	Y	Y	Y	Y	Y	Y	9/9	100
Sales et al. 2020 [32]	Y	Y	Y	Y	Y	Y	Y	Y	Y	9/9	100
Costa et al. 2020 [33]	Y	Y	Y	Y	Y	Y	Y	Y	Y	9/9	100
Resende et al. 2020 [34]	Y	Y	Y	Y	Y	Y	Y	Y	Y	9/9	100
Schneckenberg et al. 2020 [35]	Y	Y	Y	Y	Y	Y	Y	Y	Y	9/9	100
Pinto et al. 2020 [36]	Y	Y	Y	U	Y	Y	Y	Y	Y	8/9	88.8
Alvares et al. 2020 [37]	Y	Y	Y	Y	Y	Y	Y	Y	Y	9/9	100
Leal et al. 2019 [38]	Y	Y	Y	Y	Y	Y	Y	Y	Y	9/9	100
Pereira et al. 2019 [39]	Y	Y	Y	Y	Y	Y	Y	Y	Y	9/9	100
Verano et al. 2019 [40]	Y	Y	Y	Y	Y	Y	Y	Y	Y	9/9	100
Lopes et al. 2019 [41]	Y	Y	Y	Y	Y	Y	Y	Y	Y	9/9	100
Nunes et al. 2019 [42]	Y	Y	Y	Y	Y	Y	Y	Y	Y	9/9	100
Rocha et al. 2018 [43]	Y	Y	Y	Y	Y	Y	Y	Y	Y	9/9	100
Peppe et al. 2018 [44]	Y	Y	Y	Y	Y	Y	Y	Y	Y	9/9	100
Castro et al. 2018 [45]	Y	Y	Y	Y	Y	Y	Y	Y	Y	9/9	100
Prado et al. 2017 [46]	Y	Y	Y	Y	Y	Y	Y	Y	Y	9/9	100
Koettker et al. 2017 [47]	Y	Y	Y	Y	Y	Y	Y	Y	Y	9/9	100
Gama et al. 2016 [48]	Y	Y	Y	U	Y	Y	Y	Y	Y	8/9	88.8
Reis et al. 2016 [49]	Y	Y	Y	Y	Y	Y	Y	Y	Y	9/9	100
Medeiros et al. 2016 [50]	Y	Y	Y	Y	Y	Y	Y	Y	Y	9/9	100
Borges et al. 2016 [51]	Y	Y	Y	Y	Y	Y	Y	Y	Y	9/9	100
Novo et al. 2016 [52]	Y	Y	Y	Y	Y	Y	Y	Y	Y	9/9	100
Monteschio et al. 2016 [53]	Y	Y	Y	Y	Y	Y	Y	Y	Y	9/9	100
Carvalho; Brito, 2016 [54]	Y	Y	Y	Y	Y	Y	Y	Y	Y	9/9	100
Motta et al. 2016 [55]	Y	Y	Y	Y	Y	Y	Y	Y	Y	9/9	100
Monteiro et al. 2016 [56]	Y	Y	Y	Y	Y	Y	Y	Y	Y	9/9	100
Silva et al. 2015 [57]	Y	Y	Y	Y	Y	Y	Y	Y	Y	9/9	100
Pitangui et al. 2014 [58]	Y	Y	Y	Y	Y	Y	Y	Y	Y	9/9	100
Braga et al. 2014 [59]	Y	Y	Y	Y	Y	Y	Y	Y	Y	9/9	100
Vogt et al. 2014 [60]	Y	Y	Y	U	Y	Y	Y	Y	Y	8/9	88.8
Silva et al. 2013 [61]	Y	Y	Y	U	Y	Y	Y	Y	Y	8/9	88.8
Pereira et al. 2013 [62]	Y	Y	Y	Y	Y	Y	Y	Y	Y	9/9	100
Pereira; Dantas, 2012 [63]	Y	Y	Y	Y	Y	Y	Y	Y	Y	9/9	100
Pereira et al. 2012 [64]	Y	Y	Y	Y	Y	Y	Y	Y	Y	9/9	100
Enderle et al. 2012 [65]	Y	Y	Y	Y	Y	Y	Y	Y	Y	9/9	100
Salge et al. 2012 [66]	Y	Y	Y	U	Y	Y	Y	Y	Y	8/9	88.8
Koettker et al. 2012 [67]	Y	Y	Y	Y	Y	Y	Y	Y	Y	9/9	100
Silva et al. 2012 [68]	Y	Y	Y	Y	Y	Y	Y	Y	Y	9/9	100
Schneck et al. 2012 [69]	Y	Y	Y	Y	Y	Y	Y	Y	Y	9/9	100
Vogt et al. 2011 [70]	Y	Y	Y	Y	Y	Y	Y	Y	Y	9/9	100
Silva et al. 2011 [71]	Y	Y	Y	U	Y	Y	Y	Y	Y	8/9	88.8
Figueiredo et al. 2011 [72]	Y	Y	Y	Y	Y	Y	Y	Y	Y	9/9	100
Cesar et al. 2011 [73]	Y	Y	Y	U	Y	Y	Y	Y	Y	8/9	88.8
Riesco et al. 2011 [74]	Y	Y	Y	Y	Y	Y	Y	Y	Y	9/9	100
Francisco et al. 2011 [75]	Y	Y	Y	Y	Y	Y	Y	Y	Y	9/9	100
Lobo et al. 2010 [76]	Y	Y	Y	Y	Y	Y	Y	Y	Y	9/9	100
Carvalho et al. 2010 [77]	Y	Y	Y	Y	Y	Y	Y	Y	Y	9/9	100
Baracho et al. 2009 [78]	Y	Y	Y	Y	Y	Y	Y	Y	Y	9/9	100
Mouta et al. 2008 [79]	Y	Y	Y	Y	Y	Y	Y	Y	Y	9/9	100
%	100	100	100	83.1	100	100	100	100	100		

*Note:* Munn et al. 2015 [16]. Q1: Was the sample frame appropriate to address the target population? Q2: Were study participants sampled in an appropriate way? Q3: Was the sample size adequate? Q4: Were the study subjects and the setting described in detail? Q5: Was the data analysis conducted with sufficient coverage of the identified sample? Q6: Were valid methods used for the identification of the condition? Q7: Was the condition measured in a standard, reliable way for all participants? Q8: Was there appropriate statistical analysis? Q9: Was the response rate adequate, and if not, was the low response rate managed appropriately?

Abbreviations: U, Unclear; Y, Yes.

No studies were excluded based on quality assessment. Most of the included studies adequately described their study subjects and settings. The majority of studies (83.1%, *n* = 54) received “yes” responses for the evaluated questions. However, 11 studies (16.9%) (10, 25, 30, 31, 38, 50, 62, 63, 68, 73, 75) were classified as “unclear” for question 4, which addresses whether the study subjects and setting were described in sufficient detail.

### 3.2 Characteristics of the Included Studies

The publications were published between 2008 and 2023. Among the 65 selected studies, most were published in 2016, totaling nine studies (*n* = 09). Regarding the Brazilian region, the studies were predominantly conducted in the state of São Paulo (*n* = 10). Concerning the methodological approach, the studies were mostly cross‐sectional (*n* = 43), followed by descriptive studies (*n* = 16). The age of participants ranged from 10 years to over 45 years.

The study settings predominantly included maternity hospitals (*n* = 31), followed by general hospitals (*n* = 29). Data collection was conducted through interviews or medical record reviews, with sample sizes ranging from 51 (57) to 23,894 (40) women. The target population was mostly composed of women who had vaginal deliveries (*n* = 57). Statistical meta‐analysis was not feasible due to heterogeneity in study methods and sample characteristics. Table 3 summarizes the main characteristics of the studies included in this review.

**TABLE 3:** General characteristics of the included studies (*n* = 65).

Characteristics	*N* (%)
Year of publication	
2008–2011	10 (15.4)
2012–2015	14 (21.5)
2016–2019	19 (29.2)
2020–2023	22 (33.8)
Study region	
Brazil	05 (7.7)
Alagoas	01 (1.5)
Ceará	05 (7.7)
Distrito Federal	01 (1,5)
Goiás	02 (3.1)
Mato Grosso	03 (4.6)
Minas Gerais	06 (9.2)
Pará	02 (3.1)
Paraná	03 (4.6)
Pernambuco	03 (4.6)
Piauí	01 (1.5)
Rio de Janeiro	09 (13.8)
Rio Grande do Sul	07 (10.8)
Rio Grande do Norte	01 (1.5)
Roraima	01 (1.5)
Santa Catarina	04 (6.2)
São Paulo	10 (15.4)
Sergipe	01 (1.5)
Study type	
Cross‐sectional	43 (66.2)
Descriptive	16 (24.6)
Cohort	03 (4.6)
Case–control	01 (1.5)
Retrospective	02 (3.1)
Study setting^a^	
Maternity	31 (47.7)
Hospital	29 (44.6)
Birth center	08 (12.3)
Home birth	02 (3.1)
Sample size	
< 100	3 (4.6)
> 100–500	27 (41.5)
> 500–1000	12 (18.5)
> 1000–1500	7 (10.8)
> 1500–2000	3 (4.6)
> 2000–2500	—
> 2500–3000	2 (3.1)
> 3000–3500	1 (1.5)
> 3500	10 (15.4)
Target population	
Women who had a vaginal delivery	57 (87.7)
Primiparous	04 (6.2)
Adolescent	03 (4.6)
Nulliparous	01 (1.5)

*Note:* Table prepared by the authors based on published data.

^a^ The sum of the number of studies by study setting exceeds the total number of included studies (*N* = 65), considering that some studies were conducted in more than one setting.

### 3.3 Prevalence of Episiotomy in Brazil

The prevalence of episiotomy practice among women varied from 0.5% [47] to 94% [52]. Table 4 consolidates data from 65 individual studies, revealing a substantial heterogeneity in episiotomy rates across Brazil. National‐level studies indicate a prevalence ranging from 28.5% to 54.6%, with larger sample sizes often correlating with rates above 50%. Regionally, the South presents a stark contrast, particularly in Rio Grande do Sul, where recent studies (2022) continue to report rates exceeding 50%. Conversely, the Northeast region, notably the state of Ceará, generally reports lower frequencies (around 10%). The Southeast region exhibits the highest variability, ranging from 2.4% to 94%. These findings suggest that despite varying local practices, episiotomy rates in many Brazilian settings remain well above international recommendations.

**TABLE 4:** Prevalence of episiotomy among Brazilian women as reported in individual studies (*n* = 65).

Region/State	Events	Sample	%	Author/Year
National				
BR	1572	5520	28.5	Alves et al. 2021 [29]
BR	1558	4504	34.6	Gama et al. 2021 [30]
BR	12,284	23.894	51.4	Leal et al. 2019 [38]
BR	6275	11.499	54.6	Gama et al. 2016 [48]
BR	3958	7401	53.5	Leal et al. 2014 [10]
North				
PA	66	400	16.4	Costa et al. 2020 [33]
PA	60	358	16.7	Pereira et al. 2019 [39]
RR	393	4437	8.8	Nakata et al. 2022 [22]
North East				
AL	73	157	46.5	Silva et al. 2015^a^ [57]
CE	25	323	7.7	Monteiro et al. 2021^b^ [25]
CE	22	314	7	Alcântara et al. 2021 [27]
CE	23	226	10.2	Souza et al. 2020^b^ [31]
CE	7	147	4.8	Castro et al. 2018 [45]
CE	24	51	47	Motta et al. 2016 [55]
PE	166	1039	16	Pitangui et al. 2014 [58]
PE	259	2563	10	Braga et al. 2014 [59]
PE	144	495	29.1	Carvalho et al. 2010 [77]
PI	293	3004	9.7	Resende et al. 2020 [34]
RN	159	314	50.6	Carvalho; Brito, 2016 [54]
SE	200	456	43.9	Prado et al. 2017 [46]
Central‐West				
DF	214	519	41.2	Verano et al. 2019 [40]
GO	17	356	4.8	Angelim et al. 2021 [28]
GO	636	1129	56.3	Salge et al. 2012 [66]
MT	5	104	4.8	Alvares et al. 2020 [37]
MT	60	680	8.8	Medeiros et al. 2016 [50]
MT	85	103	82.5	Borges et al. 2016^a^ [51]
Southeast				
MG	140	666	21	Rodrigues et al. 2022 [21]
MG	152	577	26.3	Aguiar et al. 2020 [9]
MG	427	935	45.7	Monteiro et al. 2016 [56]
MG	236	477	49.4	Vogt et al. 2014^c^ [60]
MG	213	831	25.6	Vogt et al. 2011 [70]
MG	19	168	11.3	Baracho et al. 2009^c^ [78]
RJ	620	1515	41	Medina et al. 2023 [19]
RJ	164	352	46.6	Sales et al. 2020 [32]
RJ	115	741	15.5	Reis et al. 2016 [49]
RJ	11	458	2.4	Pereira et al. 2013 [62]
RJ	1023	155	15.2	Pereira; Dantas, 2012 [63]
RJ	218	559	39	Pereira et al. 2012 [64]
RJ	207	1287	16.1	Silva et al. 2011 [71]
RJ	50	447	11.2	Figueiredo et al. 2011 [72]
RJ	396	1359	29.1	Mouta et al. 2008 [79]
SP	517	2761	18.7	Albuquerque et al. 2022 [8]
SP	174	884	19.7	Rocha et al. 2018 [43]
SP	21	264	7.9	Peppe et al. 2018 [44]
SP	94	100	94	Novo et al. 2016^c^ [52]
SP	152	1079	14.1	Silva et al. 2013 [61]
SP	152	1019	14	Silva et al. 2012 [68]
SP	350	1316	26.6	Schneck et al. 2012 [69]
SP	1647	6365	25.9	Riesco et al. 2011 [74]
SP	184	303	60.7	Francisco et al. 2011 [75]
SP	246	958	25.7	Lobo et al. 2010 [76]
South				
PR	30	82	36.6	Schneckenberg et al. 2020 [35]
PR	27	344	7.8	Pinto et al. 2020 [36]
PR	58	154	37.7	Monteschio et al. 2016 [53]
RS	2930	5714	51.3	César et al. 2022 [11]
RS	7019	11.809	59.4	Pelissari et al. 2022 [23]
RS	2691	4521	59.5	Marmitt et al. 2022 [24]
RS	126	525	24	Domenighi et al. 2021 [26]
RS	436	746	58.4	Lopes et al. 2019 [41]
RS	241	269	89.6	Enderle et al. 2012^a^ [65]
RS	1238	1719	72	Cesar et al. 2011 [73]
SC	118	1670	7	Ferreira et al. 2023 [20]
SC	106	330	32.1	Nunes et al. 2019 [42]
SC	1	212	0.5	Koettker et al. 2017 [47]
SC	1	89	1.1	Koettker et al. 2012 [67]

*Note:* Table prepared by the authors based on published data.

Abbreviations: AL, Alagoas; BR, Brazil; CE, Ceará; DF, Distrito Federal; GO, Goiás; MG, Minas Gerais; MT, Mato Grosso; PA, Pará; PE, Pernambuco; PI, Piauí; PR, Paraná; RJ, Rio de Janeiro; RN, Rio Grande do Norte; RR, Roraima; RS, Rio Grande do Sul; SC, Santa Catarina.SE, Sergipe; SP, São Paulo.

^a^ Sample of adolescents.

^b^ Sample of nulliparous women.

^c^ Sample of primiparous women.

Regarding temporality, national‐level studies suggest a downward trend over the last decade. While earlier investigations (2014–2016) reported prevalence rates above 50%, recent national surveys from 2021 indicate a decrease, with rates ranging between 28.5% and 34.6%. However, this decline is not uniform across all regions. In Rio Grande do Sul, for instance, although there was a reduction from 72% in 2011 to approximately 51%–59% in 2022, the rates remain comparatively high. Conversely, states like Ceará demonstrate a sharper decline, with multiple studies from 2020 and 2021 reporting frequencies below 11%.

Only 16 [24.6%] reported episiotomy rates of approximately 10% or lower [20, 22, 25, 27, 28, 31, 34, 36, 37, 44, 45, 47, 50, 59, 62, 67]. A distinct subset of studies stands out for reporting episiotomy rates at or below 10%, evidencing the feasibility of restrictive practice across various Brazilian settings. The South region presented the lowest absolute rates in this review, specifically in Santa Catarina, with studies reporting frequencies as low as 0.5% and 1.1%. In the Northeast, the state of Ceará showed remarkable consistency, with multiple studies ranging between 4.8% and 10.2%. Low prevalence was also identified in the Central‐West (4.8% in Goiás and Mato Grosso) and the North (8.8% in Roraima). Even in the Southeast, a region typically marked by high interventionism, isolated studies in Rio de Janeiro (2.4%) and São Paulo (7.9%) demonstrate that specific institutions have successfully implemented restrictive protocols.

### 3.4 Factors Associated With Episiotomy in Brazil

A total of 33 (50.8%) studies analyzed the factors associated with the practice of episiotomy. Significant sociodemographic factors included adolescence or young maternal age, along with higher education and income levels. Regarding obstetric characteristics, primiparity, primigravida, nulliparity, prematurity, and macrosomia were frequently identified. Clinical management variables associated with the procedure included the use of oxytocin, supine position, and instrumental delivery. Finally, the care model was a determining factor, with higher rates observed in deliveries within the private healthcare sector and those attended by physicians. Table 5 presents a summary of the main factors associated with episiotomy.

**TABLE 5:** Factors associated with episiotomy in Brazil (*n* = 33).

Factor associated with episiotomy	References
Primiparous woman	[8, 10, 11, 20, 32, 40, 43, 54, 58, 59, 66, 68, 72, 77]
Primigravidas	[9, 23, 27, 53]
Nulliparous	[40, 44, 69, 74]
Adolescents	[23, 33, 58, 59, 66, 73, 77]
Young women	[9, 11, 42, 43, 68]
Women with higher education levels	[11, 24, 42]
Women with higher income levels	[11, 24]
Delivery in the private healthcare sector	[9, 38]
Prematurity	[20, 74]
No previous vaginal delivery	[58, 77]
Longer duration of the second stage of labor	[8]
Hospital labor lasting more than 8 h	[35]
Use of oxytocin	[11, 58, 68]
Instrumental delivery	[11, 59]
Supine position	[31, 78]
Newborn weight > 4000 g	[11, 43]
Deliveries attended by physicians	[26, 59]

*Note:* Table prepared by the authors based on published data.

The type of institution emerged as a determinant, evidencing a clear disparity between financing models. Higher rates were consistently observed in deliveries within the private healthcare sector compared to the public sector. Two studies specifically analyzed the influence of the institutional setting on episiotomy rates. These investigations, comprising a study in seven maternity hospitals in Belo Horizonte (Minas Gerais) [9], and a nationwide study involving 606 public and 12 private hospitals [38], consistently identified delivery in the private healthcare sector as a significant predictor of the procedure compared to the public sector.

## 4 Discussion

This review presented prevalence rates of episiotomy practice in Brazil, encompassing women who had vaginal births, including adolescents, primiparous, and nulliparous women, while also analyzing the most frequently associated factors. These findings may contribute to guiding future research in this field. The prevalence of episiotomy varied widely across studies, ranging from 0.5% [47] to 94% [52]. This variation likely reflects disparities in sample sizes, institutional protocols, and local cultural contexts. Notably, few studies reported rates within the WHO recommendation of approximately 10% [12], indicating that despite scientific advances, the practice remains pervasive in many Brazilian settings.

According to this review's findings, the practice of episiotomy is associated with several maternal, institutional, and clinical factors, including: primiparity, primigravidity, nulliparity, adolescence, youth, higher education, higher income, delivery in private healthcare settings, physician‐assisted births, use of oxytocin, instrumental delivery, absence of previous vaginal birth, prematurity, newborn weight over 4000 g, and horizontal delivery position.

These results are consistent with the international literature. Similar findings were observed in a study conducted in Africa, where factors associated with episiotomy included primiparity, physician‐assisted delivery, prolonged second stage of labor, use of oxytocin, instrumental delivery, and fetal macrosomia [3]. Similarly, a study in Ethiopia found a combined episiotomy prevalence of 45.11%, with main associated factors being prolonged second stage of labor, face presentation, fetal weight over 4000 g, instrumental delivery, and primiparity [4].

However, a distinct feature of the Brazilian context is the strong influence of socioeconomic and institutional variables. The association between higher socioeconomic status (higher income and education) and increased episiotomy rates reveals a paradox in the Brazilian context. Unlike in many high‐income countries where vulnerable populations receive poorer care, in Brazil, women with greater access to technology and private care are often victims of hyper‐medicalization. These women, despite being low‐risk, are exposed to unnecessary procedures under the guise of modern or safe assistance. This suggests that in the Brazilian context, access to private care does not guarantee adherence to evidence‐based practices but rather increases exposure to interventions [9, 10, 38].

This scenario highlights the persistence of the technocratic model of birth, particularly within the private sector, where physician‐assisted births are predominant. The commodification of care in these settings often drives a cascade of interventions, such as the routine use of oxytocin, lithotomy position, and episiotomy, aimed at accelerating labor to fit institutional schedules rather than respecting physiological timing [9, 10, 26, 38, 59]. This resistance to evidence‐based practices is further underscored by a study on the knowledge, attitude, and practice (KAP) of obstetric physicians in Brazil. The study revealed that inadequate KAP regarding episiotomy contributes to rates of 42%, significantly surpassing the recommended threshold [80].

In contrast, evidence consistently shows that births attended by nurse‐midwives are associated with a lower likelihood of episiotomy, reinforcing that collaborative and woman‐centered models of care are effective pathways for reducing unnecessary obstetric interventions [9, 26, 30, 48, 59]. Beyond being a clinical quality indicator, the reduction of episiotomy constitutes an ethical and human rights imperative. The performance of a surgical incision without clear clinical indication, and frequently without explicit, informed consent, may be characterized as a form of obstetric violence, representing a violation of bodily autonomy and reproductive rights [14, 15].

From this perspective, persistently high episiotomy rates reflect not only gaps in the incorporation of scientific evidence but also institutional and cultural practices that conflict with the principles of humanized childbirth. These findings underscore the urgency of strengthening public policies, professional training, and care models aligned with humanized childbirth frameworks, which emphasize respect for women's rights, informed decision‐making, and the minimization of unnecessary interventions, in accordance with national and international guidelines [12, 13].

Despite scientific evidence demonstrating the harms associated with elective episiotomy, including physical and emotional repercussions for women [81], the practice is still routinely performed in obstetric care settings worldwide. This conduct is often justified by the argument that the intervention aims to prevent adverse outcomes in women considered at high risk for complications [82]. The consequences of this practice extend beyond the immediate intrapartum period. A study conducted in Nigeria with 403 women identified that 210 of them reported post‐episiotomy morbidities. The morbidity prevalence was 52.1%, with symptoms including perineal pain lasting 72 h or more, difficulty walking, self‐reported perineal discomfort, and to a lesser extent, surgical wound infection and perineal epithelial dehiscence [83].

In light of this scenario, conservative measures during the second stage of labor are recommended before resorting to episiotomy. These strategies include optimizing perineal function through the application of warm compresses to relieve pain and stimulate local blood flow; administration of local anesthesia [84, 85]; prioritization of spontaneous pushing; and, in bed births, placing the woman in the left lateral position to reduce perineal pressure during contractions and facilitate tissue stretching [85]. Vertical birthing positions also represent an important alternative for reducing the incidence of episiotomy and perineal lacerations. Additionally, the adoption of the “hands‐on” technique, placing one hand on the fetal head and the other on the perineum, helps control descent speed and promote gradual relaxation of the perineal tissues [84, 86].

Finally, some limitations of this systematic review should be acknowledged. Meta‐analysis was not feasible due to the considerable methodological heterogeneity among included studies, as well as the presence of incomplete data in some publications. Nonetheless, strengths include the broad scope in terms of samples and settings, with no restriction on publication period, allowing the inclusion of data from diverse regions of Brazil. Moreover, this is the first systematic review to assess, at a national level, the prevalence of episiotomy and its associated factors.

## 5 Conclusion

Most studies analyzed in this review report episiotomy rates exceeding recommended benchmarks, revealing a significant gap between scientific evidence and clinical practice in Brazil. The persistence of these high rates suggests the continued influence of a technocratic model that frequently intervenes in the physiological process of childbirth, often disregarding current restrictive policies.

While sociodemographic and obstetric factors influence prevalence, the care model emerged as a decisive determinant. Evidence indicates that collaborative care involving nurse‐midwives is a protective factor against unnecessary interventions. Therefore, reducing routine episiotomy is not merely a technical adjustment but requires systemic change. It is essential to implement and enforce robust public policies and clinical protocols aligned with the principles of humanized childbirth. Strengthening the role of nurse‐midwives and ensuring that care is centered on women's rights and bodily autonomy are urgent strategies to align Brazilian obstetrics with international standards of quality and safety.

## Funding

The authors have nothing to report.

## Conflicts of Interest

The authors declare no conflicts of interest.

## Supporting information


**File S1:** birt70073‐sup‐0001‐SupplementaryFile1.docx.

## Data Availability

Data sharing not applicable to this article as no datasets were generated or analysed during the current study.
